# An Energy-Saving and Efficient Deployment Strategy for Heterogeneous Wireless Sensor Networks Based on Improved Seagull Optimization Algorithm

**DOI:** 10.3390/biomimetics8020231

**Published:** 2023-06-02

**Authors:** Li Cao, Zihui Wang, Zihao Wang, Xiangkun Wang, Yinggao Yue

**Affiliations:** 1 School of Intelligent Manufacturing and Electronic Engineering, Wenzhou University of Technology, Wenzhou 325035, China; caoli198723@163.com (L.C.);; 2 Intelligent Information Systems Institute, Wenzhou University, Wenzhou 325035, China

**Keywords:** environmental monitoring, Internet of Things, heterogeneous wireless sensor network, node deployment, seagull optimization algorithm, coverage

## Abstract

The Internet of Things technology provides convenience for data acquisition in environmental monitoring and environmental protection and can also avoid invasive damage caused by traditional data acquisition methods. An adaptive cooperative optimization seagull algorithm for optimal coverage of heterogeneous sensor networks is proposed in order to address the issue of coverage blind zone and coverage redundancy in the initial random deployment of heterogeneous sensor network nodes in the sensing layer of the Internet of Things. Calculate the individual fitness value according to the total number of nodes, coverage radius, and area edge length, select the initial population, and aim at the maximum coverage rate to determine the position of the current optimal solution. After continuous updating, when the number of iterations is maximum, the global output is output. The optimal solution is the node’s mobile position. A scaling factor is introduced to dynamically adjust the relative displacement between the current seagull individual and the optimal individual, which improves the exploration and development ability of the algorithm. Finally, the optimal seagull individual position is fine-tuned by random opposite learning, leading the whole seagull to move to the correct position in the given search space, improving the ability to jump out of the local optimum, and further increasing the optimization accuracy. The experimental simulation results demonstrate that, compared with the coverage and network energy consumption of the *PSO* algorithm, the GWO algorithm, and the basic *SOA* algorithm, the coverage of the *PSO-SOA* algorithm proposed in this paper is 6.1%, 4.8%, and 1.2% higher than them, respectively, and the energy consumption of the network is reduced by 86.8%, 68.4%, and 52.6%, respectively. The optimal deployment method based on the adaptive cooperative optimization seagull algorithm can improve the network coverage and reduce the network cost, and effectively avoid the coverage blind zone and coverage redundancy in the network.

## 1. Introduction

At present, environmental protection and monitoring are becoming more and more important, and there are many types of operating environment monitoring targets and large demand [1,2]. When using the existing traditional wired monitoring network for environmental monitoring, there will be high network construction costs, complex network node deployment, future maintenance will be difficult, and other problems [3,4]. The mature development and wide application of Wireless Sensor Networks (WSNs) technology will provide more reliable and efficient technical support for environmental monitoring [5]. WSNs have the characteristics of low power consumption, strong robustness, self-organization, and scalability. It can realize large-scale deployment in target areas, ensure the breadth and accuracy of data collection, reduce the cost of monitoring network construction, and reduce network deployment and maintenance costs [6,7]. The difficulty of post-maintenance can effectively meet the needs of large-scale deployment of environmental monitoring and long-term online monitoring. According to whether the node structure, function and link are the same, it can be divided into homogeneous wireless sensor networks (WSNs) and heterogeneous wireless sensor networks (HWSNs) [8,9]. Compared with homogeneous WSNs, HWSNs can better reflect the heterogeneity and polymorphism of the physical world, so it is easier to achieve a synchronous perception of multiple targets with the collaborative execution of multiple tasks, and its application range is becoming wider and wider [10].

The heterogeneous wireless sensor network is an advanced form of wireless sensor network that is not only compatible with various key technologies of the homogeneous network but also utilizes the heterogeneous characteristics of nodes to improve network performance [11]. Combining the optimal coverage algorithm into the heterogeneous network environment can better meet the different needs of practical applications. The core issues that need to be solved urgently in heterogeneous wireless sensor networks–network coverage, energy consumption, and network connection reliability are closely related [12,13]. Network coverage determines the ability of wireless sensor networks to monitor the physical world, reflecting the “perceived” quality of service that the network can provide [14].

Due to the limited energy of nodes and the heterogeneous characteristics of the network itself, the redundancy of sensing data and the timeliness of aggregation, the data collection method of heterogeneous sensor networks has become one of the key and difficult issues in the current sensor network research field. Construct a heterogeneous sensor network QoS support system framework that meets the quality-of-service requirements such as reliability, connectivity, fault tolerance, and energy efficiency, and propose an efficient coverage control algorithm oriented to application requirements and heterogeneous characteristics. Reasonably utilize the limited resources in the wireless sensor network, reduce the energy consumption of sensor nodes, improve the life cycle and service quality of the network, optimize the deployment location of wireless sensors, and maximize the coverage of the target area.

### 1.1. Problem Description and Research Motivation

Node deployment technology is a key technology of heterogeneous wireless sensor networks, which has an important impact on the performance of sensor networks [15]. According to the deployment method, node deployment is divided into deterministic deployment and random deployment. Deterministic deployment refers to a deployment technology in which node location information or network status is known before deployment [16]. How to ensure the connectivity and coverage performance of the network in a deterministic deployment method is favored by researchers [17].

Although the current research on the coverage problem of sensor networks has achieved certain results, most of them ignore the problem of network connectivity, which is not only to ensure that the object is monitored and perceived but also to transmit the monitored data to the background for further monitoring. It should also be noted that the sensors used in engineering practice have different parameters, such as sensing radius, communication radius, and cost, due to different manufacturing processes or types (such as temperature sensors, humidity sensors, infrared sensors, etc.). Most of the existing research results assume that the sensor network is composed of nodes with the same parameters, and there are great limitations in applying these research results to sensor networks composed of nodes with different parameters. Furthermore, sensor network nodes are often deployed in harsh working environments, and the failure rate of nodes is high due to energy depletion, physical environment, and man-made damage. Ensuring fault tolerance of the network and providing reliable monitoring services is very important for the target-oriented monitoring sensor network. Finally, most of the above research results assume that the cost of nodes in the area is the same when deploying nodes and do not take into account the different deployment costs caused by differences in deployment locations.

### 1.2. Contribution

Compared with the traditional method, the particle swarm collaborative optimization seagull algorithm proposed in this paper comprehensively considers issues such as energy consumption, the network’s lifetime, and the network coverage. The main contributions of this paper:

1. While proposing the coverage control algorithm problem, we described the coverage control method problem of HWSNs.
2. We proposed a coverage deployment strategy based on the particle swarm collaborative optimization seagull algorithm.
3. We performed a large number of simulation calculations to improve the efficiency and coverage of the network coverage optimization algorithm.
4. We evaluated the superior performance of the proposed algorithm by comparing it with the algorithms of *PSO*, GWO, and *SOA*.

## 2. Related Work

The heterogeneity of HWSNs is reflected in node heterogeneity, link heterogeneity, and network protocol heterogeneity. The problem of network coverage refers to how to ensure that the coverage formed by the deployed nodes meets the requirements of the monitoring environment. Network coverage is usually divided into two types: deterministic coverage and random coverage.

### 2.1. Deterministic Coverage

Deterministic coverage is subdivided into how to achieve the maximum coverage with a fixed number of nodes rate and how to achieve the target coverage with the least number of nodes. Liu et al. consider a new problem in rechargeable wireless sensor networks, given a set of locations with one or more targets, to determine the minimum number of sensor nodes that need to be deployed to ensure a given coverage quality [18]. Karimi et al. modeled the heterogeneous node deployment problem as an optimization problem with the total power consumption of the network as its cost function and proposed a heterogeneous two-layer Lloyd algorithm to optimize node deployment and improve network coverage [19]. Djenouri et al. [20] considered the communication coverage of sustainable data forwarding in wireless sensor networks and proposed an energy-aware deployment model for relay nodes. Compared with existing heuristic algorithms, the running time of the proposed heuristic algorithm is shorter. Due to the limited node energy and high node mobility performance consumption, it is necessary to reduce energy consumption through the node deployment process. In order to solve these problems, Liu et al. [21] proposed a distributed node deployment algorithm DABVF based on virtual forces to improve the network coverage of underwater wireless sensor networks. How to effectively deploy all wireless sensors and save system energy consumption is a key issue in current wireless sensor network applications. Deng et al. [22] proposed an optimization strategy by adding an external central force to deploy sensors from the center of the target area to avoid covering holes or abnormal structures.

### 2.2. Random Coverage

Random coverage refers to the optimization of reducing energy consumption of nodes and prolonging the life cycle of the network by adjusting the state switching of nodes after the implementation of uniform distribution of nodes in a specific environment. Priyadarshi et al. [23] divided various coverage technologies into four main parts: computational geometry-based technology, force-based technology, grid-based technology, and meta-heuristic-based technology. Akram et al. [24] used different parameters to verify the consistency of the method used for scheduling sensor nodes, allowing it to cover all targets while using less power. The experiment proved that this method used an effective method for scheduling sensor nodes to monitor all targets with less power consumption. Bhat et al. [25] proposed a localization and deployment model that uses arithmetic optimization algorithms (AOA) for localization and node deployment, and further utilized the results of this algorithm to develop deployment models to achieve fully connected networks. Tirandazi et al. [26] proposed a new algorithm that improves connectivity and full area coverage by utilizing some mobile robots to change the initial topology and position of sensors. The proposed algorithm has shown significant improvements in connectivity and area coverage. Narayan et al. [27] proposed a coverage optimization and vulnerability repair protocol to optimize the overlap and coverage vulnerability problems in the network and maximize coverage. Using probability theory and statistical theory, they established the relationship between the coverage area and the radius of SN. This protocol improves the stability period of the network and improves the network coverage. In the literature [28,29,30,31], the author uses swarm intelligence optimization algorithms, such as particle swarm optimization, artificial bee colony algorithm, artificial fish swarm algorithm, shark optimization algorithm, and other methods to transform the problem into a combinatorial optimization problem. It is good to employ the high-performance optimization capability of swarm intelligence optimization algorithms and improve the connectivity of wireless sensor networks.

In response to these problems, the characteristics of numerous deployment nodes, low network connectivity and target coverage performance, and poor fault tolerance in target monitoring applications are considered. In this paper, how to deploy sensor nodes with different performance parameters in a given deployment location with different costs, so that the cost optimization problem under the condition of multiple coverage of monitoring targets and multiple connectivity between deployed sensor nodes is studied and proposed. A minimum-cost fault-tolerant deployment strategy for heterogeneous sensor networks is proposed for target monitoring. By constructing the mathematical model of connectivity and coverage of heterogeneous sensor nodes, the optimization problem is solved with the help of the improved Seagull optimization algorithm, and the coverage and connectivity performance of the sensor network are guaranteed under the condition of optimal deployment cost. In heterogeneous wireless sensor networks, there are problems of high node deployment density, poor target coverage and network connectivity fault tolerance, and high deployment cost. A cost-optimized fault-tolerant deployment strategy for node heterogeneous sensor networks based on particle swarm collaborative optimization seagull algorithm is proposed.

## 3. Mathematical Model

For the two-dimensional sensor network coverage problem in HWSNs, it is assumed that there are *n* monitoring target points to be covered in the deployment area of the two-dimensional plane, and the deployed sensor nodes adopt isomorphic sensors, that is, the sensing radius of the sensors is the same [32,33]. Let the sensing radius be *R_s_*, the communication radius be *R_c_*, the unit of the network coverage is *m*, and 2*R_s_* ≤ *R_c_*. Assuming that there are *n* monitoring target points in the area to be covered and setting the position coordinates of the *i*-th sensor node as (*x_s_*, *y_s_*), the Euclidean distance of the target point to be monitored that the sensor node can cover is shown in Formula (1):

(1) $$d(i,s)=\sqrt{{\left(x_{s}-x_{i}\right)}^{2}+{\left(y_{s}-y_{i}\right)}^{2}}$$

The probability *p*(*i*, *s*) of the target point *i* to be monitored while being covered by the sensor node *s* is shown in Formula (2):

(2) $$p(i,s)=\left\{\begin{array}{l} 0,d(i,s)>R_{s}\\ 1,d(i,s)\leq R_{s} \end{array}\right.$$

The two-dimensional planar area to be deployed is equally divided along the *x* and *y* axes with a step size *q*, then the length of each segment is *l* = *q*, and the intersection point of the deployment area grid is *q*^2^. Then, the probability that the monitoring point set *T* in the deployment area is sensed by the sensor node set *Q*, the node coverage rate, is shown in Formula (3).

(3) $$Cov=\frac{p_{\mathrm{cov}}}{q^{2}}=\frac{\sum_{i=1}^{S}p(i,s)}{q^{2}}$$

The number of sensors in the node set *Q* is *S*.

**Figure 1:**
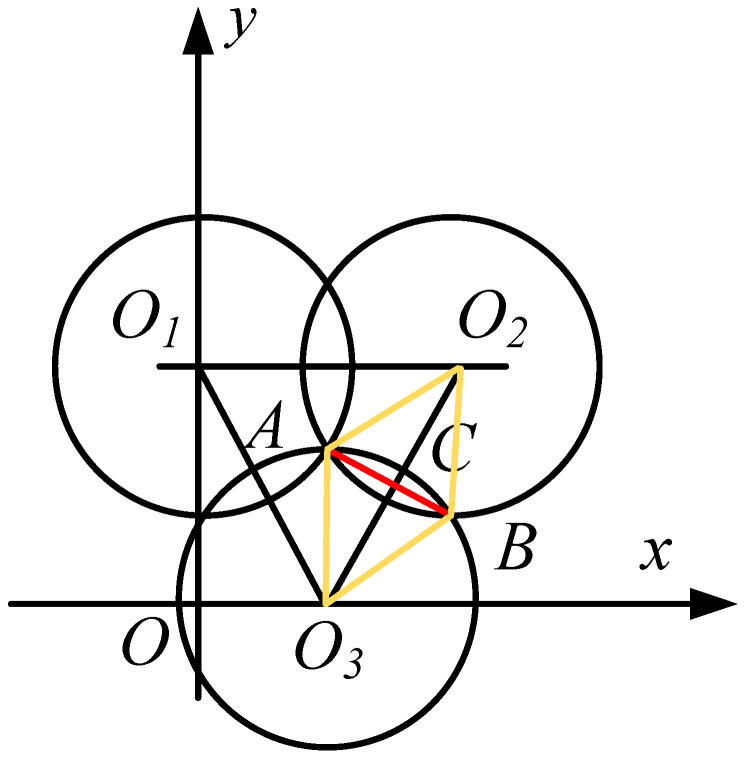
The sensor node coverage diagram.

Suppose the deployment area is a square with side length *L*. Theoretically, in the set deployment area, the number of deployment nodes can be obtained by calculation, and the schematic diagram of the full coverage deployment of nodes is shown in Figure 1.

In Figure 1, *O*_1_, *O*_2_, and *O*_3_, respectively, represent the positions of the three sensor nodes. At this time, the triangle *O*_1_*O*_2_*O*_3_ is an equilateral triangle, and *O*_3_*A* = *R_s_* is the sensing radius of the sensor. If ∠*AO*_3_*B* = π/3, *AB* = *BO_3_* = *O*_3_*A* = *R_s_*, according to the nature of the circle, then *AB*⊥*O*_2_*O*_3_, ∠*AO*_3_*C* = 1/2∠*AO_3_B* = π/6. According to the cosine theorem, the length of the line segment *O*_3_*C* is shown in Formula (4).

(4) $$L_{O_{3}C}=L_{O_{3}A}\times \cos(∠AO_{3}C)=R_{s}\times \cos(\pi /6)=(\frac{\sqrt{3}}{2})R_{s}$$

Theoretically, the number of nodes in the deployment area is shown in Formula (5):

(5) $$M={\left(\frac{L}{(\sqrt{3}/2)\times R_{s}+R_{s}}+1\right)}^{2}$$

According to the above analysis, the node deployment problem can be simplified as a constrained engineering optimization problem, as shown in Formula (6):

(6) $$s.t.\left\{\begin{array}{l} g_{1}=\sum_{i=1}^{S}p(i,s)\geq 0\\ g_{2}=\sum_{i=1}^{S}p(i,s)-q^{2}\\ g_{3}=d(i,s)-R_{s}\geq 0\\ g_{4}=S-M\geq 0 \end{array}\right.$$

Based on the situation of energy balance in the network, the definition of energy balance coefficient *η* is introduced, in detail: the parameter *E_i_* is used to define the remaining energy of sensor node *i*, and the parameter *k* is the number of valid sensor nodes.

(7) $$\eta =\frac{Max(E_{i})-Min(E_{i})}{\frac{1}{k}\sum_{i=1}^{k}E_{i}}$$

The parameter *η* reflects the balance of network energy consumption. The more balanced the network energy consumption is, the lower the network energy consumption, and the smaller the value.

## 4. Seagull Optimization Algorithm Optimized by *PSO* (*PSO-SOA*)

### 4.1. Seagull Optimization Algorithm (*SOA*)

Seagull optimization algorithm (Seagull optimization algorithm, *SOA*) is a new type of intelligent optimization algorithm based on mathematical models established by Gaurav et al., based on the migration and attack behavior of seagulls [34]. The algorithm has many advantages [35,36,37], including but not limited to simple structure, fewer parameters, and lower time complexity, but at the same time, it also has disadvantages such as weak global optimization ability, low population diversity, and falls easily into local optimum [38,39]. There are also many advantages. In the seagull optimization algorithm, a mathematical model for global exploration of the algorithm is established for long-distance migration, and a mathematical model for local development is established for spiral attacks [40].

#### 4.1.1. Migration (Global Exploration)

The seagull optimization algorithm simulates the movement trajectory of seagull flocks in the air when they are flying. When it changes position, the following conditions need to be met [41]:

(a) Avoid collisions. To prevent collisions with other individual seagulls during flight, the seagull optimization algorithm uses an additional control variable of inertia weight *A* to update the position of a single seagull.

(8) $$C_{i}\left(t\right)=A\times X_{i}\left(t\right)$$

Among them, *C_i_* is the non-collision part, and *t* is the repeated number. $X_{i}\left(t\right)$ is the update times of the position $X_{i}\left(t\right)$. The inertia weight *A* is the behavior of the seagull in the specified space, and the calculation formula is:

(9) $$A=f_{c}-\left(t\times \frac{f_{c}}{MaxIter}\right)$$

Among them, MaxIter is the maximum number of iterative updates, and *t* is the current number of iterations. The initial value of $f_{c}$ is a constant of two, which is used to control the change in *A* value with the number of iterative updates. As the number of updates increases, *A* linearly decreases from the initial value $f_{c}$ to 0.

(b) Adjacent to each other. Under the control of *A*, individual seagulls will approach the nearest seagull with the best fitness value under the premise of preventing collision with other individual seagulls.

(10) $$M_{i}\left(t\right)=B\times \left(X_{best}\left(t\right)-X_{i}\left(t\right)\right)$$

(11) $$B=2\times A^{2}\times rand$$

Among them, $M_{s}\left(t\right)$ indicates that the seagull is moving in the best direction. $X_{best}$ indicates the best position of the seagull. *B* represents the random factor that controls *A* in order to balance the exploration and development of the algorithm, and rand represents the value number of the random (0, 1).

(c) Approach the position of the optimal seagull until it arrives. Individual seagulls are updated relative to the optimal seagull position.

(12) $$D_{i}\left(t\right)=\left|C_{i}\left(t\right)+M_{i}\left(t\right)\right|$$

Among them, $D_{i}\left(t\right)$ is the interval between the seagull and the best seagull at the iteration number *t*. In summary, the seagull population avoids collisions while increasing the search area, and then moves to the optimal seagull.

#### 4.1.2. Attack (Partial Exploitation)

It is our purpose to make more use of the experience in the search process [42]. After the seagull arrives in a new area, it will use its own advantage to rush towards the prey’s speed and angle to make a spiral attack when it is preying on the prey. The calculation method of the spiral attack is shown by the vectors *x*, *y*, *z*.

(13) $$r=u\times e^{\theta v}$$

(14) $$x^{\prime }=r\times \cos\left(\theta \right)$$

(15) $$y^{\prime }=r\times \sin\left(\theta \right)$$

(16) $$z^{\prime }=r\times \theta$$

Among them, the parameter *r* is the radius of the spiral movement during the attack. θ is the random value of $[0,2\pi ]$. *u* and *v* represent constants (take one in the paper), and the parameter *e* is the natural logarithm base.

(17) $$X_{i}\left(t\right)=\left(D_{i}\left(t\right)\times x^{\prime }\times y^{\prime }\times z^{\prime }\right)+X_{best}\left(t\right)$$

Wherein, $X_{i}\left(t\right)$ is the new position of the seagull after the attack [43].

### 4.2. Implementation Process of Seagull Optimization Algorithm Optimized by PSO (PSO-SOA)

The particle swarm optimization algorithm is a more convenient and efficient intelligent algorithm discovered and proposed by Kennedy et al. [44]. Particle swarm optimization simulates birds in a flock of birds by designing a massless particle. The particle has only two attributes: speed and position. The speed represents the speed of movement, and the position represents the direction of movement. Each particle searches for the optimal solution independently in the search space, records it as the current individual extremum, shares the individual extremum with other particles in the entire particle swarm, and finds the optimal individual extremum as the entire particle, the current global optimal solution of the group. The core idea of this algorithm is that all individuals in the particle swarm will move towards the two optimal directions during each iteration [45]. Additionally, based on this, it iteratively updates its position several times, and there is no guarantee of global optimization when dealing with the *PSO* [46,47]. Current researchers are more focused on improving and optimizing *SOA* based on the two aspects of individual population and optimal position, and do not pay attention to the optimization of the worst position of seagulls. However, when implementing the *SOA* algorithm, the distribution of the worst position of seagulls in the entire space will affect the optimization of the entire iterative process, so the optimization of its worst position can make it closer to the optimal position, thereby improving the optimization speed of the entire algorithm [48].

#### 4.2.1. Particle Swarm Optimization Algorithm with Jump Operator

In the later stage of the iteration of the particle swarm algorithm, the optimal coordinates will be limited to the local area, making it difficult to jump out of the local extremum. Therefore, an adaptive jump operator is added in the iterative process to compare the similarity between the optimal coordinates of individual particles and the optimal coordinates of group particles, giving particles different jump probabilities. After the *N*-th iteration, the probability formula and jump formula of the *i*-th particle jumping out of the current position are:

(18) $$\begin{array}{c} p=\exp\left(f\left(p_{gbest}^{N}\right)-f\left(p_{i}^{N}\right)\right); \end{array}$$

(19) $$\begin{array}{c} x_{i}^{N}=x_{i}^{N}+rand\times \left(ub-lb\right); \end{array}$$

Among them: the parameter *rand* is a random number between [0, 1]. The parameters *ub* and *lb* are upper and lower limits. It can be obtained from the formula that when the optimal position of the particle is close to the optimal position of the population, the particle has a greater probability of jumping out, so as to avoid falling into the local optimal situation.

#### 4.2.2. Seagull Optimization Algorithm Optimized by *PSO* (*PSO-SOA*)

Based on the particle swarm optimization algorithm, this paper takes the global optimal position of *SOA* and any position whose fitness is better than the worst position and sets these two positions as two “extreme” positions, and then distributes them uniformly. The worst position of individual seagulls is close to the direction of the other two positions. The expression is shown in Formula (20). Finally, based on the calculation of Formula (20), it is determined whether the worst position of the seagull needs to be replaced by a position with better fitness.

(20) $$\begin{array}{r} X_{temp}=X_{worst}^{t}\times rand+\left(X_{worst}^{t}-X_{ri}^{t}\right)\times rand\\ +\left(X_{worst}^{t}-X_{best}^{t}\right)\times rand \end{array}$$

(21) $$X_{worst}^{t+1}=\left\{\begin{array}{l} X_{temp}\;\;\;\;\;f\left(X_{temp}\right)<f\left(X_{i}^{t}\right)\\ X_{i}^{t}\;\;\;\;\;\;\;\;\;otherwise \end{array}\right.$$

In Formulas (20) and (21), $X_{temp}$ represents the intermediate temporary position after being processed, $X_{best}^{t}$ is the optimal seagull position, $X_{worst}^{t}$ is the worst seagull position, and $X_{ri}^{t}$ is any position of the seagull population. The essence of this strategy is to intersect the global optimal position and the global worst position, make the worst position move towards the optimal position, and combine any position to make it approach any position better than itself. In the iterative process of the *SOA* algorithm, the original worst-case position will be replaced by any improved position that is better than it. Under such iterative calculation, continuous optimization of seagull individuals can be achieved, which helps the algorithm accelerate its optimization speed.

### 4.3. Algorithm Complexity Analysis

Assuming that the number of populations of the algorithm is *N*, the dimension of the search space is *D*, and the maximum number of searches is *T_max_*, the complexity of *PSO-SOA* includes: the initialization complexity of the population *O*(*ND*), the fitness value calculation complexity *O*(*ND*), the position update complexity of the global and local search is *O*(*N*^2^log*D*), the fitness value sorting complexity of the algorithm is *O*(*N*^2^), and the control parameter update complexity of the algorithm is O(*ND*). Then, the complexity of the *PSO-SOA* algorithm is shown in Formula (22):

(22) $$O(PSO-SOA)=O(ND)+O(T_{\max})O(ND+N^{2}\log N+N^{2}+ND)$$

The algorithm time complexity of *SOA* is shown in Formula (23):

(23) $$O(SOA)=O(ND)+O(T_{\max})O(N^{2}\log N+N+ND)$$

## 5. Node Deployment Strategy of HWSNs Based on the *PSO-SOA* Algorithm

**Figure 2:**
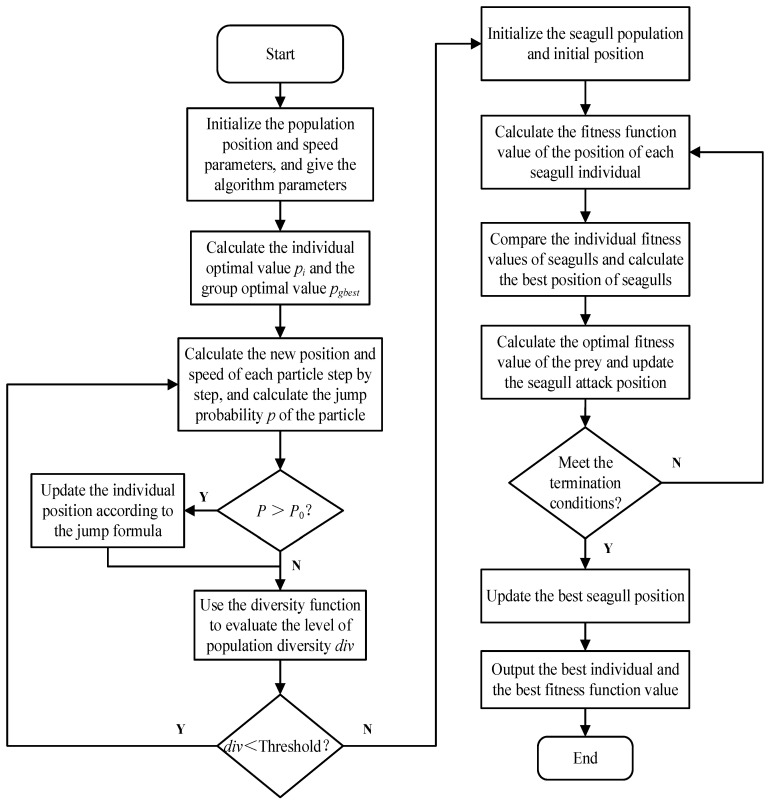
Flow chart of *PSO-SOA* algorithm.

The improved seagull algorithm, to solve the sensor node deployment optimization problem of the heterogeneous sensor network, firstly, is aimed at the initialization of the HWSNs system and seagull algorithm. The information of each individual seagull is updated through group learning of the best individuals after screening out the best individuals of the individual seagulls, including the spatial position, coverage, etc. The initial population is generated by the chaotic initialization method so that the overall convergence speed of the seagull algorithm is improved. In addition, due to the poor convergence speed and search ability of the seagull algorithm, the *PSO* algorithm is used to improve the overall search ability. When iteratively optimizing, the seagull community movement law is used to cooperate with individuals, attract each other, and conduct information interaction. It is necessary to obtain the optimal solution to obtain better convergence speed and the optimal individual. The improved Seagull algorithm will speed up the convergence, the diversity of the population will increase, and the coverage of HWSNs will also be improved in the subsequent search process. To design the optimal coverage strategy of HWSNs based on the particle swarm optimization seagull algorithm, one needs to solve the maximum value of the objective function of the coverage optimization for HWSNs, obtain the distribution positions of all sensor nodes in the area to be tested after optimal deployment, and output the optimized coverage rate. The algorithm flow chart of the seagull algorithm based on particle swarm optimization is shown in Figure 2.

The specific experimental steps are as follows:

(1) Set the coverage model parameters of the sensor nodes in HWSNs, and generate and calculate the sensor node positions that initialize the corresponding network coverage according to the objective function. Set the total number *N* of the initial gull population and the maximum number of iterations *T_max_*;
(2) Calculate fitness. Each individual seagull is evaluated based on its position. The initialization of individual optimal coverage *P_best_* and population optimal coverage *P_g_* for each individual seagull;
(3) Calculate the position of the seagull after the collision through the migratory behavior of the individual seagull, and move to the optimal individual and calculate the relative displacement position;
(4) Calculate the position of the spiral attack according to Formulas (13)–(16);
(5) Use the *PSO* mechanism to update the worst seagull individual position, and calculate the worst seagull individual position according to Formulas (20) and (21);
(6) According to the average optimal position of the calculated population, the position of each individual seagull is updated, and the coverage rate of HWSNS of each individual seagull position after the update is calculated according to the objective function *f*;
(7) Compare the individual coverage rate of each seagull after updating the position, corresponding to the coverage rate of the individual optimal *P_best_*, if the former is larger, the *P_best_* will be updated;
(8) Perform backtracking iterative update, update the seagull population and update the optimal coverage, and output the optimal solution found;
(9) If the cycle does not reach the preset maximum number of iterations, return to 2); otherwise, it ends, and the optimal solution is output.

## 6. Algorithm Simulation Comparison and Analysis

### 6.1. Simulation Environment Settings

In order to reflect the superior performance of the Particle Swarm Optimization Seagull Algorithm (*PSO-SOA*) proposed in this paper, we need to accurately test and improve the performance of the *SOA* algorithm, and when simulating various algorithms, to ensure the fairness of the comparison algorithm conditions and set consistent parameters. At the same time, the proposed *PSO-SOA* algorithm is compared with particle swarm optimization algorithm (*PSO*) [49], gray wolf optimization algorithm (GWO) [50], and seagull algorithm (*SOA*), and its performance is tested by a unimodal function and a multimodal function. Additionally, a large number of experiments are carried out on the coverage performance of HWSNS to verify the superior performance of the algorithm proposed in this paper. Each algorithm was run 50 times in the simulated parameter settings of HWSNS coverage to obtain an average value reflecting its coverage performance. In total, 40 sensor nodes were set to be randomly deployed in a monitoring area of 100 × 100 m^2^, and the initial energy of each node was 1 J. Considering that the heterogeneity of HWSNS is mainly reflected in the sensing range of the nodes, we set the sensing radius of each sensor node as a random number within a range of [5,15]. In order to compare the performance of the analysis algorithms, the initial positions of the sensor nodes were randomly generated in the monitored area, using consistent simulation conditions. The experimental operating environment was IntelCoreTMI7-8700CPU, 3.2 GHz, 32 GB memory, 64-bit operating system Windows10, and the simulation software used MATLABR2020B.

### 6.2. Test Objective Function Optimization

In order to verify that the improved *PSO-SOA* algorithm has better performance in terms of convergence and optimization speed, this paper conducted comparative experiments on the basis of the six test functions in Table 1 for the standard test function information. If the algorithm ran to the maximum number of iterations, the algorithm terminated the optimization problem for the selected six functions. In order to avoid randomly affecting the experimental results, the three algorithms independently conducted 200 experiments on each optimization problem and recorded the average of the 200 running results.

Table 2 presents the experimental results obtained by independently running *PSO-SOA*, *SOA*, GWO, and *PSO* algorithms on multiple test functions 200 times.

**Figure 3:**
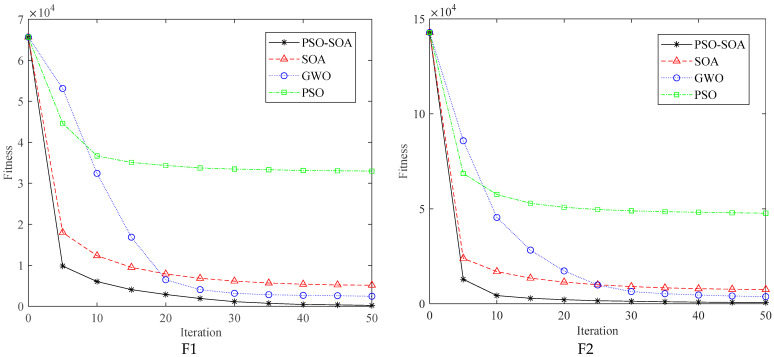

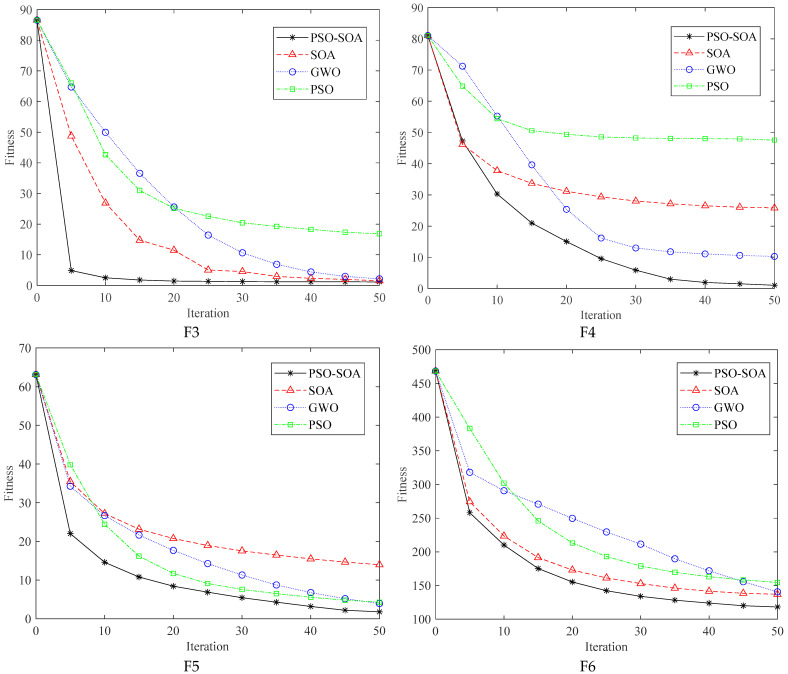
Comparison of convergence curves for function optimization.

Table 2 lists the optimal values, average values, and standard deviations obtained by independently running *PSO-SOA*, *SOA*, GWO, and *PSO* algorithms 200 times. It can be seen that for the selected test functions, *PSO-SOA* has the strongest optimization performance, which is obviously better than *SOA*, GWO, and *PSO*. For function F4, the *PSO-SOA* algorithm improves accuracy by two compared with the basic *SOA* algorithm, and the algorithm as a whole has better stability. For functions F2 and F3, the optimal value of the *PSO-SOA* algorithm is close to 0, but in terms of stability, the optimization performance of *PSO-SOA* is much better than that of *SOA* and GWO. The effect is relatively poor, and it can be seen that the improved algorithm has obvious competitive advantages in performance. In order to visually demonstrate the optimization performance of *PSO-SOA*, we selected six benchmark functions such as the convergence iteration curve shown in Figure 3.

From the convergence curves of the above six test function optimizations, it can be seen that the improved *PSO-SOA* algorithm is better than the *PSO*, GWO, and basic *SOA* algorithms in terms of convergence speed. The functions of F1 and F3 are unimodal functions, in which it is easier to achieve the optimum, so they are mostly used to examine the connection ability. The improved *PSO-SOA* algorithm has higher convergence precision, which overcomes the problem of low precision of basic *SOA* optimization, which can be seen intuitively from the function convergence curve. At the same time, it can be seen from the convergence curve that the improved algorithm can easily jump out of the local optimum and converge to the optimal value at a faster speed, and there are multiple inflection points in the iterative process. Compared with the basic *SOA* algorithm, the improved *PSO-SOA* algorithm improves the accuracy of optimization. For the multimodal functions F5 and F6, the improved *PSO-SOA* algorithm has a faster convergence speed than *PSO*, GWO, and basic *SOA* algorithm and finds the best optimal solution with the best performance. As mentioned above, it can be known from the convergence curve that the convergence of the *PSO-SOA* algorithm is better than the basic *PSO*, GWO, and *SOA* algorithms.

### 6.3. Simulation Results and Analysis

#### 6.3.1. Comparison of Coverage Effects of Sensor Node Deployment

**Figure 4:**
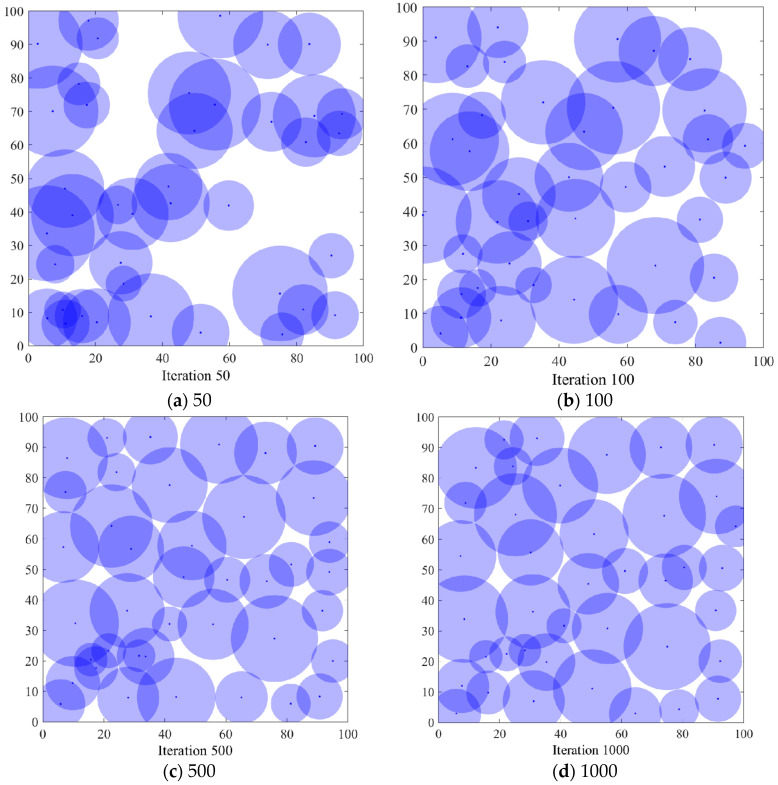
Coverage effect of *PSO* algorithm.

**Figure 5:**
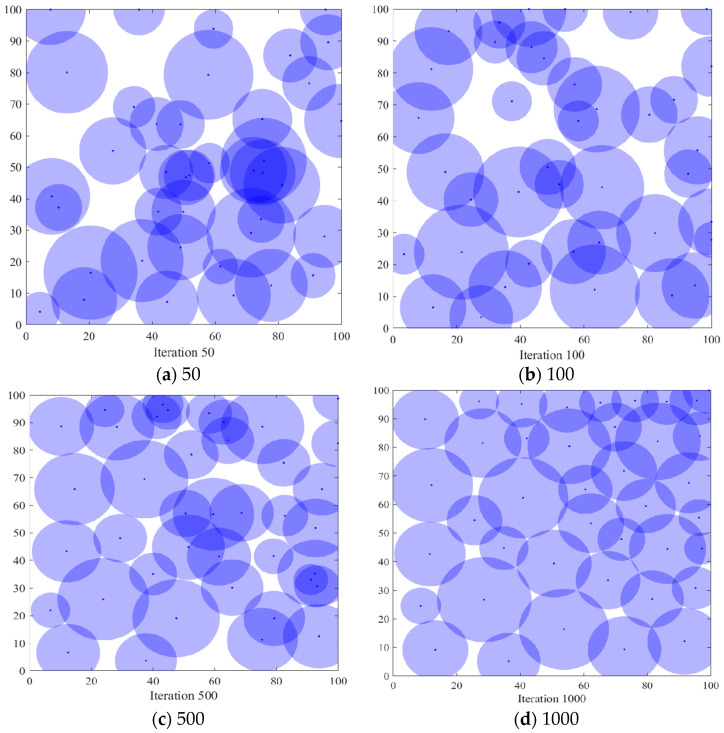
Coverage effect of GWO algorithm.

**Figure 6:**
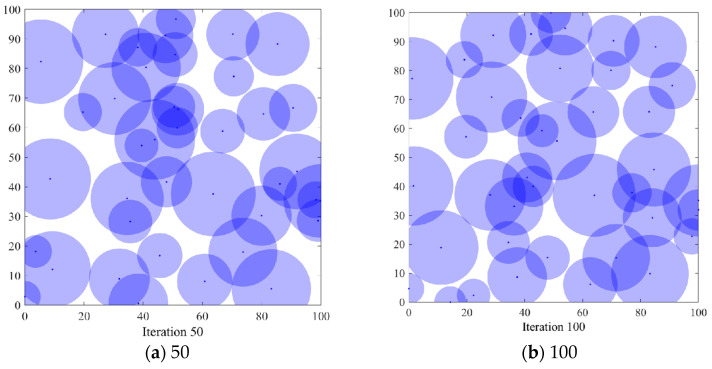

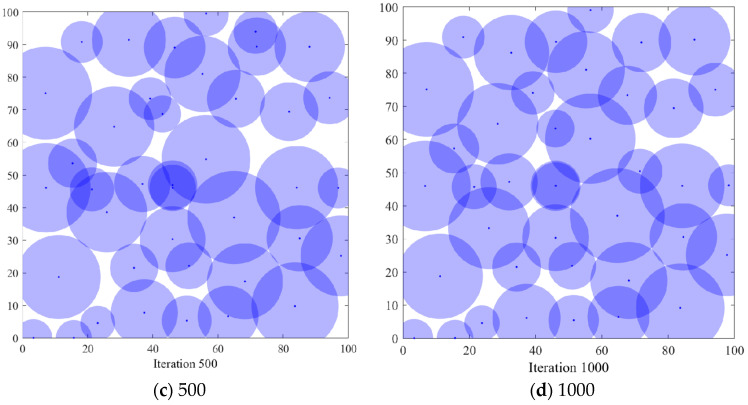
Coverage effect of *SOA* algorithm.

**Figure 7:**
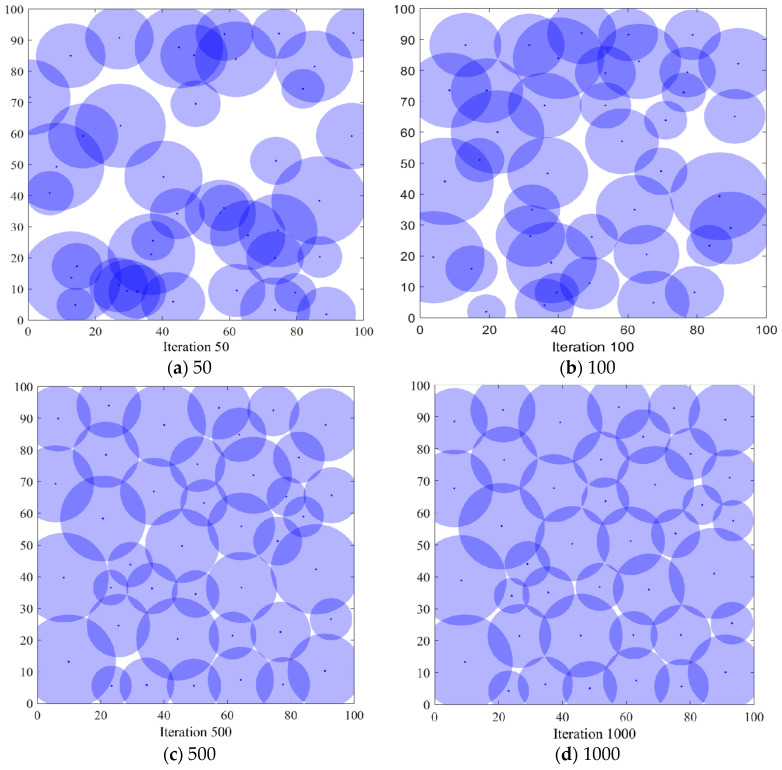
Coverage effect of *PSO-SOA* algorithm.

In order to verify the coverage effect of the algorithm *PSO-SOA* proposed in this paper, the optimal coverage simulation results of the three algorithms of the GWO algorithm and the basic *SOA* algorithm under different iterations from the *PSO* algorithm are shown in Figure 4, Figure 5, Figure 6 and Figure 7 below. Figure 4 is the simulation coverage effect of the *PSO* algorithm, Figure 5 is the simulation coverage effect of the GWO algorithm, Figure 6 is the simulation coverage effect of the *SOA* algorithm, and Figure 7 is the simulation coverage effect of the *PSO-SOA* algorithm proposed in this paper.

From Figure 4, Figure 5, Figure 6 and Figure 7, it can be seen that the coverage of the four algorithms’ HWSNS increases with the increase in the number of iterations, and the increase is relatively large. From the perspective of 1000 iterations, the coverage effect of HWSNs of *PSO* algorithm is poor, the coverage effect of HWSNs of GWO algorithm is average, the coverage effect of HWSNs of *SOA* algorithm is better, and the coverage effect of HWSNs of *PSO-SOA* algorithm is the best. The network coverage effects of the four algorithms are superimposed when the algorithm is iterated 100 times. However, at 1000 iterations, the *PSO-SOA* algorithm proposed in this paper has the least overlapping coverage of different nodes, the *PSO* algorithm has the most overlapping coverage of nodes, the GWO algorithm has more overlapping coverage of nodes, and the *SOA* algorithm has more overlapping coverage. The overlapping parts of the coverage of the nodes of the algorithm are less. In this paper, the *PSO-SOA* algorithm is proposed to improve the node work efficiency and reduce the network cost, and at the same time, the coverage effect is the best.

#### 6.3.2. Comparison of Network Coverage with Different Number of Nodes

**Figure 8:**
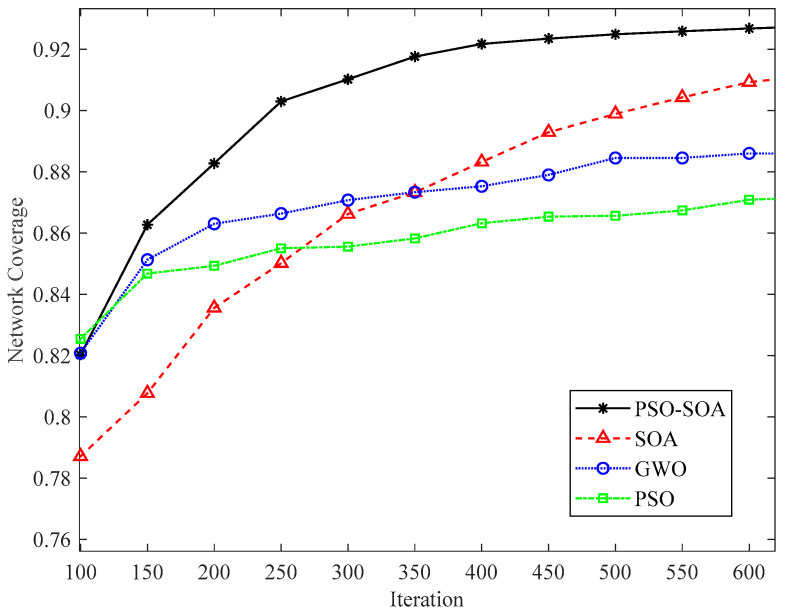
Coverage effect of 40 nodes.

**Figure 9:**
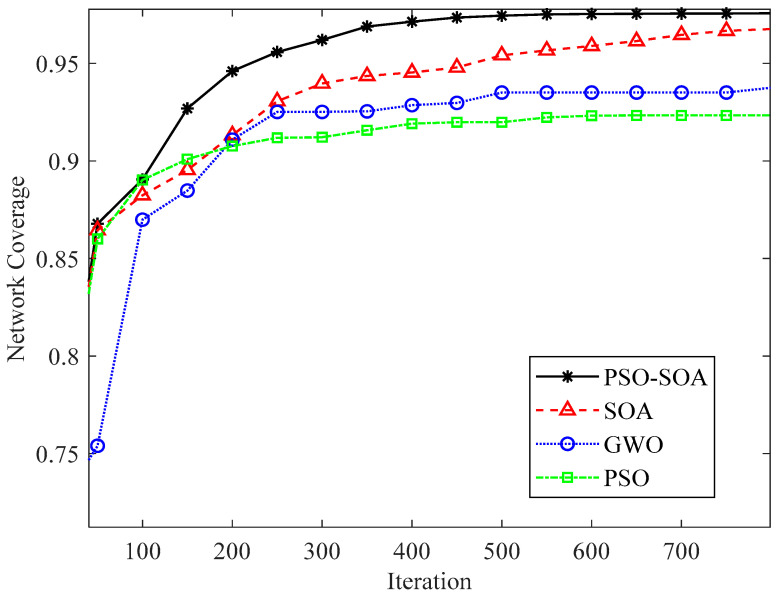
Coverage effect of 50 nodes.

**Figure 10:**
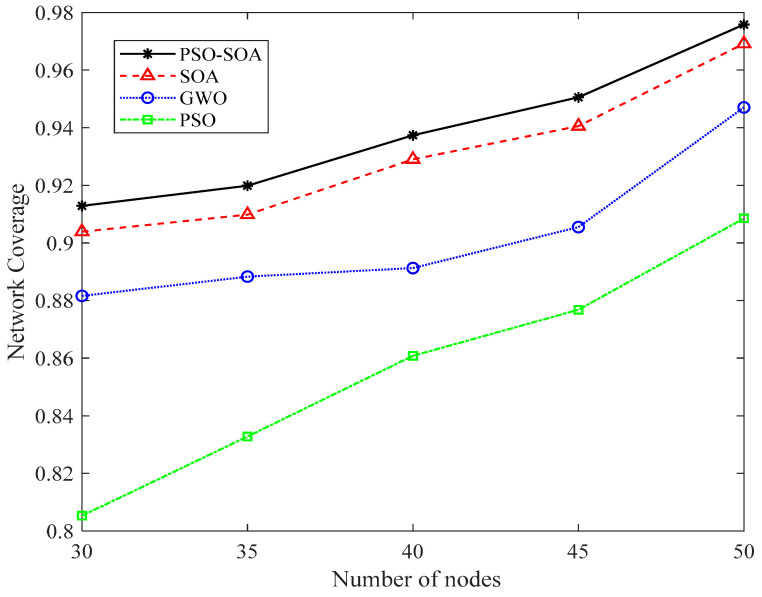
Comparison of network coverage under different number of nodes.

We increased the network coverage effect in the case of different numbers of nodes to increase the experimental effect. Figure 8 and Figure 9 show the network coverage effect of 40 and 50 sensor nodes in the case of 1000 iterations. In addition, we compared the 40 and 50 nodes covered by the network, as shown in Figure 10. It can be seen from Figure 8, Figure 9 and Figure 10 that the network coverage of the four algorithms gradually increases with the increase in the number of iterations. The 100-iteration network coverage of the four algorithms is a reference, the network coverage of 40 nodes is above 0.68, and the network coverage of 50 nodes is above 0.72. After 1000 iterations, the network coverage of 40 nodes is above 0.58, and the network coverage of 50 nodes is above 0.92. In addition, it can be seen from Figure 8, Figure 9 and Figure 10 that no matter whether the number of nodes is 40 or 50, the network coverage rate of the *PSO* algorithm is the lowest, the network coverage rate of the GWO algorithm is low, and the network coverage rate of the *SOA* algorithm is high. It is proposed that the *PSO-SOA* algorithm has the highest network coverage. While improving the working efficiency of nodes and reducing network costs, the *PSO-SOA* algorithm proposed in this paper improves the coverage effect of nodes. Taking the number of sensor nodes as 40 in Figure 8 as an example, the network coverage rate of the *PSO* algorithm is 87.8%, the GWO algorithm is 89.1%, the *SOA* algorithm is 92.9%, and the *PSO-SOA* algorithm proposed in this paper is 93.9%. Compared with the coverage rates of the *PSO* algorithm, the GWO algorithm and the basic *SOA* algorithm, the *PSO-SOA* algorithm proposed in this paper improves over them by 6.1%, 4.8%, and 1.2%, respectively.

#### 6.3.3. Comparison of 3D Energy Consumption

**Figure 11:**
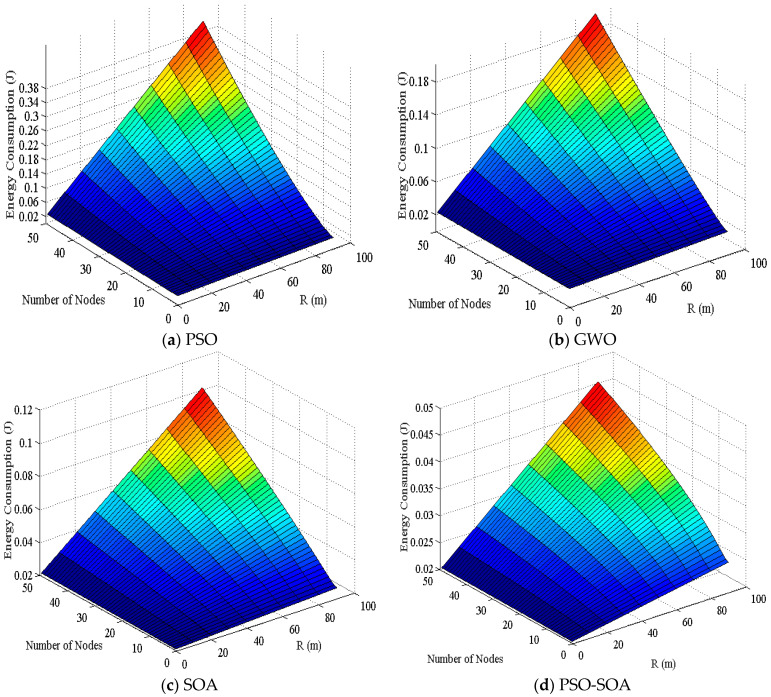
Comparison of 3D Energy Consumption.

The problem of network energy consumption has always been a hot topic in HWSNS research, and it is also a problem that everyone is very concerned about, which also restricts the progress of network coverage research. By deploying reasonable nodes into the network, coupled with node sleep/wake control, power control, etc., the energy utilization rate of the network is maximized, and at the same time, energy balance is achieved for optimal network coverage performance. Figure 11 shows the comparison of network three-dimensional energy consumption of the four algorithms proposed in this paper: *PSO*, GWO, *SOA*, and *PSO-SOA*.

It can be seen from Figure 11 that the network energy consumption of the four algorithms is relatively small when the number of sensor nodes and the communication radius are the same. At the same time, it can be seen from the four figures that the energy consumption of the network gradually increases with the increase in the number of nodes and the increase in the communication radius. Among them, the *PSO* algorithm has the largest energy consumption in the network, and the energy consumption of most nodes is relatively high, and the energy consumption is uneven, reaching a maximum of 0.38 J. The energy consumption of the GWO algorithm is also relatively large, and the energy consumption of most nodes is also relatively high, and the energy consumption is also relatively balanced. The highest energy consumption of the sensor nodes reaches 0.18 J. The network energy consumption of the basic *SOA* algorithm is relatively small, and the energy consumption of network nodes can reach up to 0.12 J. The *PSO-SOA* algorithm proposed in this paper has the smallest network energy consumption, the energy consumption of sensor nodes is more balanced, and the energy consumption of network nodes can reach up to 0.02 J. Overall, the energy consumption of the *PSO* algorithm is the largest, the energy consumption of the GWO algorithm is high, the network energy consumption of the basic *SOA* algorithm is small, and the average energy consumption of the *PSO-SOA* algorithm proposed in this paper is the smallest. Compared with the network energy consumption of *PSO* algorithm, GWO algorithm, and basic *SOA* algorithm, the *PSO-SOA* algorithm proposed in this paper reduces 86.8%, 68.4%, and 52.6%, respectively, over them.

**Figure 12:**
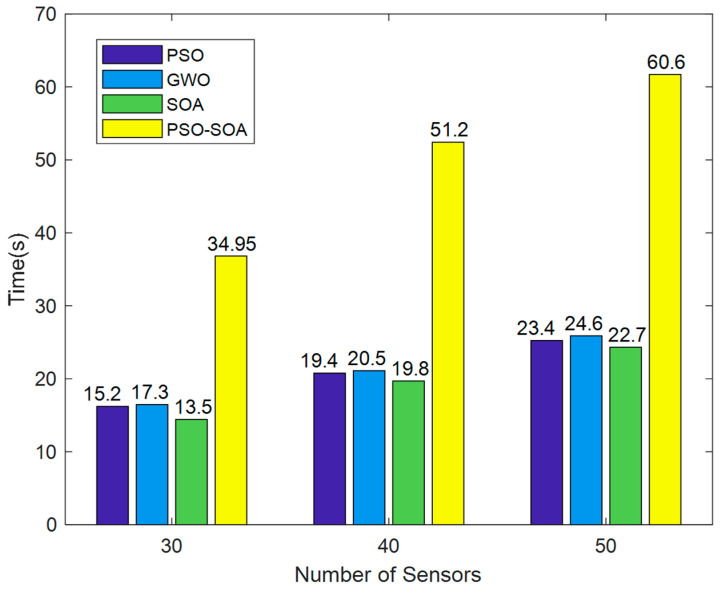
Algorithm simulation time comparison.

Simulation time of the algorithm: The calculation speed of the response algorithm is also one of the important indicators of network performance. In the case of 30, 40, and 50 sensor nodes, the simulation time comparison chart of the four algorithms is shown in Figure 12.

It can be seen from Figure 12 that the network of the four algorithms takes a short time when the number of sensor nodes is 40, and the *PSO* algorithm takes the shortest time, which is 20.77 s. The *PSO-SOA* algorithm proposed in this paper takes 51.2 s, and the *SOA* algorithm takes 19.8 s. When the network of four algorithms has 50 sensor nodes, the required time is relatively long, mainly due to the increase in the number of nodes, the intelligent optimization algorithm with increased calculation and the relatively increased simulation time. The *PSO-SOA* algorithm proposed in this paper takes 60.6 s, and the simulation time is relatively long. This is the problem we will solve next.

Based on the above simulation results, it can be seen that since the scaling factor is introduced into the *PSO-SOA* algorithm proposed in this paper, the relative displacement between the current seagull individual and the optimal individual has been dynamically adjusted so that the diversity of the seagull algorithm has been improved. Enhanced, the overall optimization capability and convergence speed have been improved. It performs well in the optimized deployment of heterogeneous wireless sensor nodes and has the best coverage effect. Therefore, the *PSO-SOA* algorithm proposed in this paper has strong adaptability and faster optimization speed, and an algorithm that can significantly improve network coverage and reduce coverage blind spots is applied to the HWSNS coverage optimization problem.

## 7. Conclusions

The random deployment of nodes in heterogeneous wireless sensor networks causes a large amount of coverage redundancy, resulting in energy wastage. In this paper, a particle swarm collaborative optimization seagull algorithm-based algorithm (*PSO-SOA*) for the optimal coverage method of heterogeneous sensor networks is proposed, with network coverage and reduction in network energy consumption as the main optimization objectives. The proposed algorithm has changed the previous problem of simply relying on intelligent algorithm optimization to solve the problem of coverage optimization. In the face of large-scale heterogeneous network coverage, from the perspective of geometric figures, a simple and efficient heterogeneous coverage optimization algorithm is proposed. The algorithm idea is simple, and the complexity is low. Additionally, coverage is fast and efficient. The algorithm can achieve ideal network coverage with a small number of iterations. Compared with other intelligent optimization and comparison algorithms, the network node coverage is significantly improved. As another optimization goal, the average energy consumption of network nodes is also significantly reduced. In summary, the algorithm is significantly better than the comparative intelligent optimization algorithm in terms of convergence speed, network coverage, and algorithm energy consumption and is especially suitable for network coverage of large heterogeneous wireless sensor networks.

Although the *PSO-SOA* algorithm proposed in this paper improves the coverage performance and work efficiency of the HWSNs network, some regional nodes are too clustered during the application process. The future research direction should be to make the coverage of HWSNS more uniform, and the area where nodes gather should be less. At the same time, the mobile sensor network coverage is considered, and the next work will mainly study the optimal coverage of the mobile sensor network.

## Figures and Tables

**Table 1:** Basic information of test functions.

Function	Formula	Dimension	Bounds	Optimum
Sphere	$F01=\sum_{i=1}^{d}x_{i}^{2}$	30	[−100, 100]	0
Step	$F02=\sum_{i=1}^{d}{\left(\left|x_{i}+0.5\right|\right)}^{2}$	30	[−100, 100]	0
Quartic	$F03=\sum_{i=1}^{d}ix_{i}^{4}+rand\left(0,1\right)$	30	[−1.28, 1.28]	0
Alpine	$F04=\sum_{i=1}^{d}\left|x_{i}\sin\left(x_{i}\right)+0.1x_{i}\right|$	30	[−10, 100]	0
Rastrigin	$F05=10d+\sum_{i=1}^{d} [x_{i}^{2}-10\cos\left(2\pi x_{i}\right)]$	30	[−5.12, 5.12]	0
Ackley	$F6=-20\exp\left(-0.2\sqrt{\frac{1}{d}\sum_{i=1}^{d}x_{i}^{2}}\right)-\exp\left(\frac{1}{d}\sum_{i=1}^{d}\cos\left(2\pi x_{i}\right)\right)+20+\exp\left(1\right)$	30	[−32, 32]	0

**Table 2:** Experimental results of function testing.

Function	*PSO-SOA*			*SOA*			GWO			*PSO*		
	Max	Mean	Std	Max	Mean	Std	Max	Mean	Std	Max	Mean	Std
F1	1.98 × 10^2^	3.21 × 10^2^	2.19 × 10^2^	4.89 × 10^3^	5.24 × 10^3^	4.79	3.04 × 10^4^	4.87 × 10^4^	5.24 × 10^3^	2.27 × 10^2^	5.87 × 10^2^	3.04
F2	5.97 × 10^1^	1.41 × 10^2^	2.87	2.98 × 10^3^	3.05 × 10^3^	7.07 × 10^1^	8.28 × 10^3^	9.05 × 10^3^	6.34 × 10^2^	1.97 × 10^2^	2.03 × 10^2^	2.34
F3	1.15	4.07	3.09	1.27	1.95	3.01 × 10^−1^	1.57 × 10^1^	5.98 × 10^1^	2.84 × 10^1^	3.02	2.87 × 10^2^	4.27 × 10^2^
F4	2.23	5.12	1.74	1.23 × 10^1^	1.16 × 10^1^	1.35 × 10^−1^	4.04	5.86	1.48	4.07	6.87	3.04
F5	1.32 × 10^2^	1.24 × 10^2^	1.98 × 10^1^	1.58 × 10^2^	1.82 × 10^2^	1.64 × 10^−1^	1.31 × 10^2^	1.56 × 10^2^	1.64 × 10^1^	1.57 × 10^2^	2.34 × 10^2^	6.01 × 10^1^
F6	2.65	3.21	2.07 × 10^−1^	1.29 × 10^1^	1.53 × 10^1^	3.24 × 10^−3^	5.48	4.57	2.12 × 10^−1^	3.05	4.12	4.01 × 10^−1^

## Data Availability

The data used to support the findings of this study are available from the corresponding authors upon request.
